# Crystal structures of two erbium(III) complexes with 4-amino­benzoic acid and 4-chloro-3-nitro­benzoic acid

**DOI:** 10.1107/S2056989015020319

**Published:** 2015-11-07

**Authors:** Graham Smith, Daniel E. Lynch

**Affiliations:** a Science and Engineering Faculty, Queensland University of Technology, GPO Box 2434, Brisbane, Queensland 4001, Australia; b Exilica Limited, The Technocentre, Puma Way, Coventry CV1 2TT, England

**Keywords:** crystal structure, erbium complexes, 4-amino­benzoic acid, 4-chloro-3-nitro­benzoic acid, coordinating dimethyl sulfoxide, hydrogen bonding

## Abstract

The crystal structures of two erbium(III) complexes with 4-amino­benzoic acid (4-ABAH), namely bis­(μ_2_-4-amino­benzoato-κ^2^ *O*:*O*′)bis­[bis(4-amino­benzoato-κ^2^ *O*,*O*′)di­aqua­erbium(III)] dihydrate, [Er_2_(C_7_H_6_NO_2_)_6_(H_2_O)_4_]·2H_2_O, (I), and 4-chloro-3-nitro­benzoic acid (CLNBAH), namely poly[hexa­kis­(μ_2_-4-chloro-3-nitro­benzoato-κ^2^ *O*:*O*′)bis­(dimethyl sulfoxide-κ*O*)dierbium(III)], [Er_2_(C_7_H_3_ClNO_4_)_6_(C_2_H_6_OS)_2_]_*n*_, (II), have been determined. In the structure of solvatomorphic compound (I), the symmetry-related irregular ErO_8_ coordination polyhedra in the discrete centrosymmetric dinuclear complex comprise two monodentate water mol­ecules and six carboxyl­ate O-atom donors, four from two bidentate carboxyl­ate *O*,*O*′-chelate groups and two from the bis-monodentate *O*:*O*′-bridging group of the third 4-ABA anion. The Er—O bond-length range is 2.232 (3)–2.478 (3) Å and the Er⋯Er separation in the dinuclear complex unit is 4.7527 (4) Å. One of the coordinating water mol­ecules is involved in an intra-unit O—H⋯O hydrogen-bonding association with an inversion-related carboxyl­ate O-atom acceptor. In contrast, the anhydrous compound (II) is polymeric, based on centrosymmetric dinuclear repeat units comprising ErO_7_ coordination polyhedra which involve four O-atom donors from two bidentate *O*:*O*′-bridging carboxyl­ate groups, one O-atom donor from the monodentate dimethyl sulfoxide ligand and two O-atom donors from the third bridging CLNBA anion. The latter provides the inter-unit link in the one-dimensional coordination polymer extending along [100]. The Er—O bond-length range in (II) is 2.239 (6)–2.348 (6) Å and the Er⋯Er separation within the dinuclear unit is 4.4620 (6) Å. In the crystal of (I), extensive inter-dimer O—H⋯O and N—H⋯O hydrogen-bonding inter­actions involving both the coordinating water mol­ecules and the solvent water mol­ecules, as well as the amine groups of the 4-ABA anions, give an overall three-dimensional network structure. Within this structure are also weak π–π ring inter­actions between two of the coordinating ligands [ring-centroid separations = 3.676 (3) and 3.711 (2) Å]. With (II), only weak intra-polymer C—H⋯O, C—H⋯Cl and C—H⋯S inter­actions are present.

## Abstract 2

In the structures of two Er^III^ compounds with 4-amino­benzoic acid and 4-chloro-3-nitro­benzoic acid, discrete centrosymmetric bridged dinuclear complex units are present giving an overall three-dimensional hydrogen-bonded structure in the first complex and a one-dimensional coordination polymer in the second.

## Chemical context   

The coordination chemistry of the rare earth (*RE*) metals has been investigated extensively and the structures of a large number of complexes with various ligand types are known (Sastri *et al.*, 2003 ▸). Of inter­est is the lanthanide contraction across the series and 4-amino­benzoic acid (4-ABAH) has provided a valuable ligand for this purpose in a comprehensive study of this effect with the *RE* ^3+^ (La–Y) series of complexes (Sun *et al.*, 2004 ▸). Within this series there are two sub-sets of isotypic complexes, one monoclinic (*P*2_1_/*n*) (La–Tb as well as Dy and Er), in which the structures are two-dimensional, the second triclinic (*P* 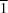) forming dinuclear structures (Yb, Lu, Y, as well as Tb). The solvatomorphism of the Tb member {monoclinic, [Tb_2_(4-ABA)_6_(H_2_O)_2_]; triclinic [[Tb_2_(4-ABA)_6_(H_2_O)_2_]·2H_2_O]} is of inter­est and its occurrence was indicated as being dependent on pH control in the preparation.
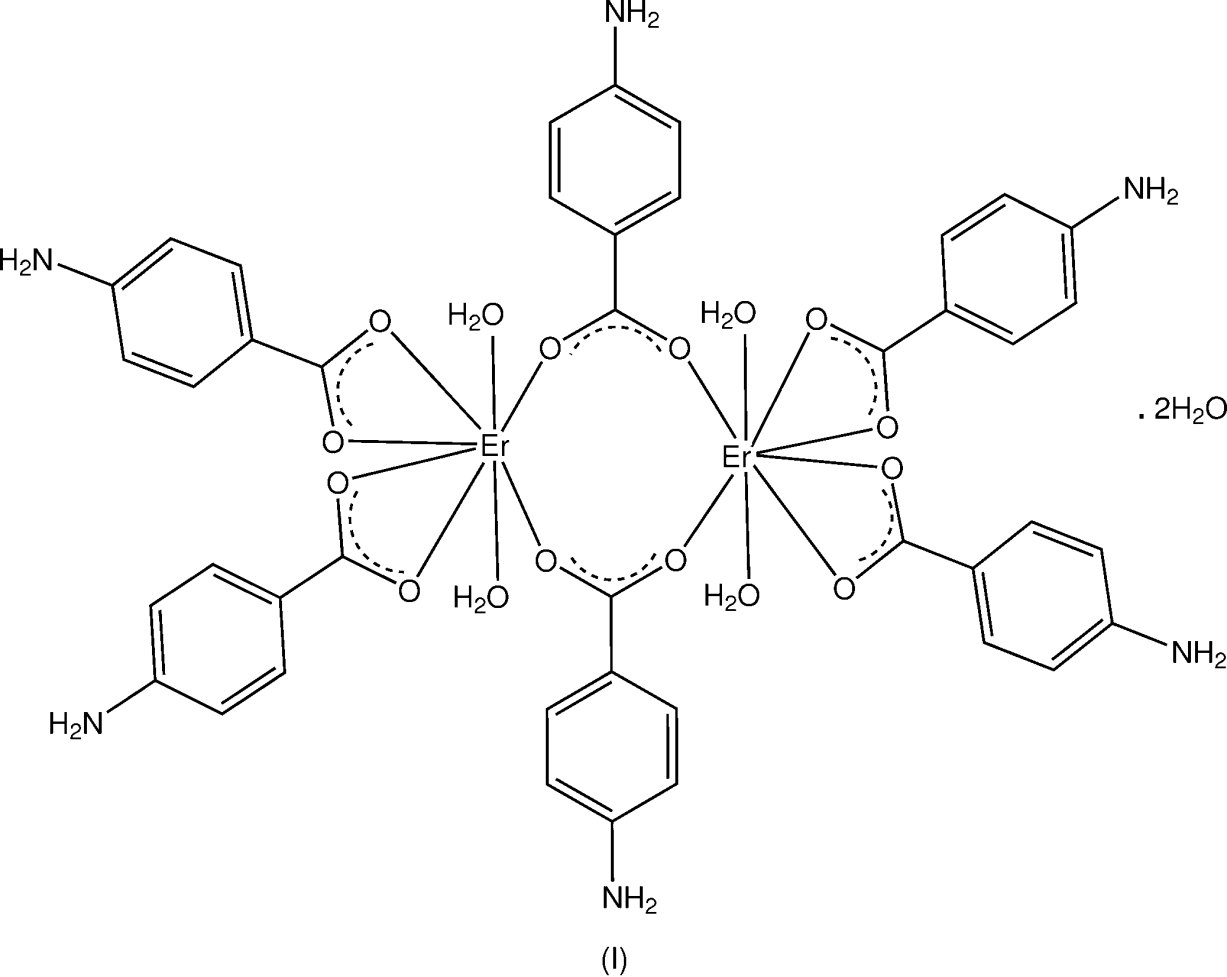
 
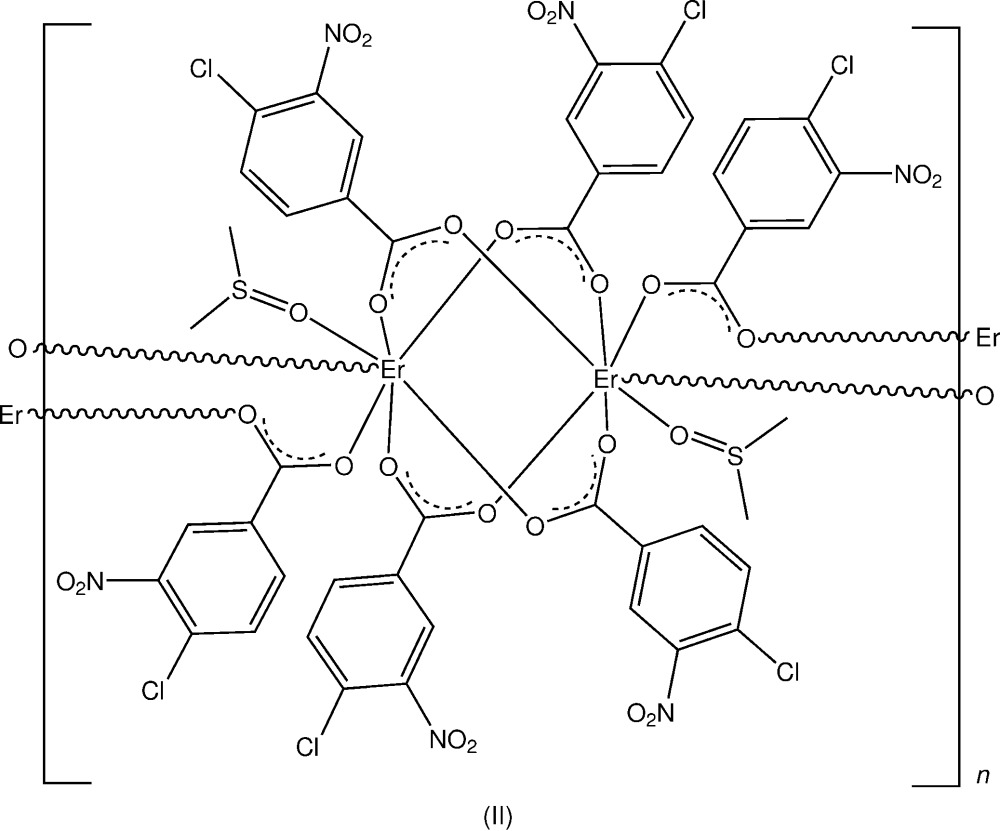


It was considered that some of the other later members of the *RE* series (predominantly triclinic) might also show the same effect so this was tested with Er in a reaction of erbium(III) acetate with 4-ABA in aqueous ethanol under mild reaction conditions, with no additional pH control. The title triclinic complex [Er_2_(C_7_H_6_NO_2_)_6_(H_2_O)_4_]·2H_2_O, (I), was obtained. For (I), the preliminary unit-cell data (Table 1 ▸) suggested a possible solvatomorphic variant of the previously reported polymeric monoclinic Er^3+^ complex with 4-ABA (Sun *et al.*, 2004 ▸), and this was confirmed in the X-ray structural analysis. The comparative cell data for the triclinic Tb^3+^ complex with 4-ABA are *a* = 9.0964 (1), *b* = 11.0117 (1), *c* = 12.7430 (2) Å, α = 89.372 (5), β = 72.0360 (6), γ = 75.0730 (7)°, *V* = 1169.97 (2) Å^3^, confirming that the two are isotypic.

Complex (II), anhydrous [Er_2_(C_7_H_3_ClNO_4_)_6_(C_2_H_6_OS)_2_]_*n*_, was obtained in a similar reaction to (I), using erbium(III) acetate and 4-chloro-3-nitro­benzoic acid (CLNBAH), with subsequent recrystallization using DMSO. The structures of both complexes are reported herein.

## Structural commentary   

**Figure 1:**
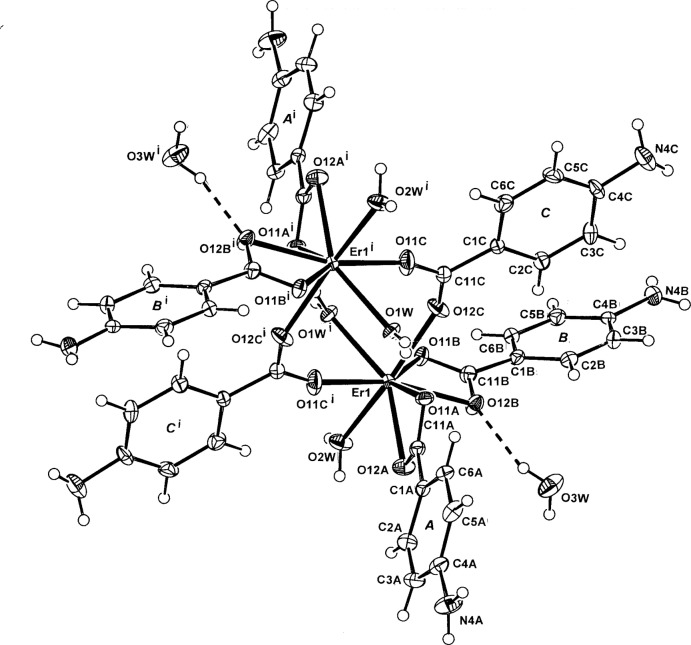
The mol­ecular configuration and atom-naming scheme for the centrosymmetric dinuclear title complex and water mol­ecules of solvation in (I), with displacement ellipsoids drawn at the 40% probability level. For symmetry code (i), see Table 1 ▸.

In the title centrosymmetric dinuclear structure of compound (I) (Fig. 1 ▸), the two identical irregular ErO_8_ complex units [Er—O bond length range, 2.232 (3)–2.478 (3) Å] (Table 1 ▸), comprise two monodentate water mol­ecules (O1*W*, O2*W*), four O-atom donors from two slightly asymmetric bidentate *O,O*’ chelate carboxyl­ate groups (the *A* and *B* 4-ABA ligands) and two bridging O-atom donors from two symmetry-related ligands (*C*). The Er⋯Er^i^ separation in the dinuclear unit is 4.7527 (4) Å. Unlike the polymeric solvatomorphic Er^III^ complex [Er_2_(4-ABA)_6_(H_2_O)_2_]_*n*_·*n*H_2_O (Sun *et al.*, 2004 ▸), in which the extending Er—N bond is somewhat elongated at 2.660 (3) Å, with (I), there is no reasonable Er—N bonding contact. The monodentate water mol­ecule O2*W* in (I) replaces the bridging amino N-donor site which is present in the 8-coordination sphere about Er in the solvatopolymorph. Within the dinuclear complex unit of (I), an intra-dimer O—H⋯O_carboxyl­ate_ hydrogen bond is present between one of the the coordinating water mol­ecules (O1*W*) and an inversion-related carboxyl­ate O-atom (O11*A* ^i^) (Table 2 ▸). This structure is similar to the triclinic isotypic Tb^3+^ complex with 4-ABA (Sun *et al.*, 2004 ▸).

In (I), the 4-ABA ligand species show some variation in the conformation of the carboxyl­ate groups. In one of the bidentate *O,O′*-chelate ligands (*A*) and the bridging ligand (*C*), the groups are essentially coplanar with the benzene ring [torsion angles C2*A/C*—C1*A/C*—C11*A/C*—O11*A/C* = 171.2 (4) and 174.8 (4)°, respectively], while in the second bidentate chelate ligand (*B*) the group is rotated out of the plane [corresponding torsion angle = 155.9 (4)°]. Such a ’planar’ conformation is also found in the structure of the parent acid (Gracin & Fischer, 2005 ▸) and in mol­ecular adducts with aromatic carb­oxy­lic acids (Chadwick *et al.*, 2009 ▸).

**Figure 2:**
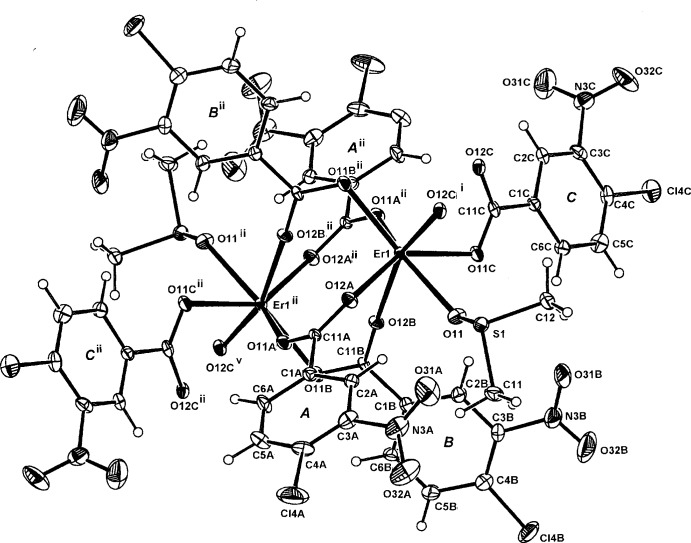
The mol­ecular configuration and atom-naming scheme for the centrosymmetric dinuclear repeat unit in the polymeric complex (II), with displacement ellipsoids drawn at the 40% probability level. [Symmetry code: (v) *x* + 1, *y*, *z*; for other symmetry codes, see Table 3 ▸.]

**Figure 3:**
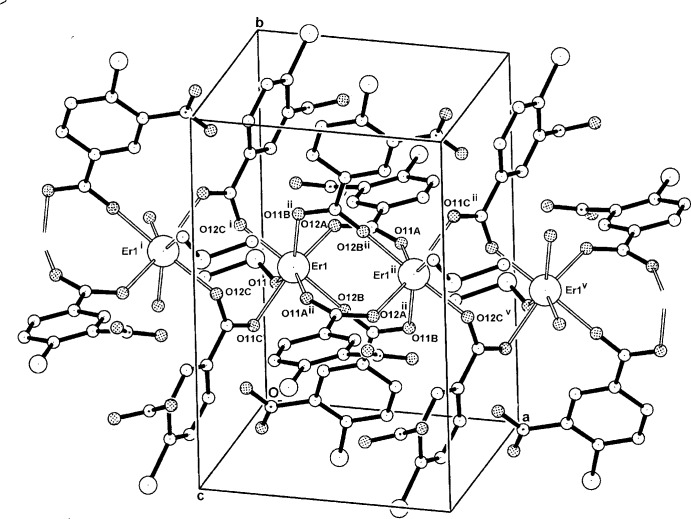
The packing of the one-dimensional polymeric chain structure of (II) in the unit cell, viewed approximately along [001]. H atoms have been omitted.

In the crystal structure of complex (II), a centrosymmetric dinuclear repeat unit is present with the two inversion-related Er^III^ atoms (Fig. 2 ▸) being seven-coordinated through four bridging carboxyl­ate *O,O* ^1^ groups (the *A* and *B* ligands), a monodentate DMSO O-atom and O-donors (O12*C* ^i^) and O11*C* ^i^ from the *C* ligand which extends the dinuclear unit into a one-dimensional coordination polymer lying along [100] (Fig. 3 ▸). The Er—O bond length range is 2.239 (6)–2.348 (6) (Table 3 ▸) and the Er⋯Er^ii^ separation within the dimeric unit is 4.4620 (6) Å. Also present within the repeat unit are a C2*B*—H⋯O11 hydrogen bond [3.298 (13) Å] and a C2*A*—H⋯S1 inter­action [3.743 (10) Å] (Table 4 ▸).

The torsion angles defining the conformation of the carboxyl­ate groups of the CLNBA ligands in (II) are C2*A/B/C*—C1*A/B/C*—C11*A/B/C*—O11*A/B/C* = 158.7 (9), 177.2 (9) and 160.3 (8)°, respectively. The torsion angles of the nitro groups C2*A/B/C*—C3*A/B/C*—N3*A/B/C*—O32*A/B/C* are −150.4 (12), 174.1 (16) and 120.3 (13)°, respectively. In the structure of the parent CLNBAH acid (Ishida & Fukunaga, 2003 ▸), the corresponding torsion angles are 174.02 (17) and −132.61 (18)° compared to 179.7 (2) and −137.8 (2)° in the Na–CLNBA monohydrate salt (Smith, 2013 ▸).

## Supra­molecular features   

**Figure 4:**
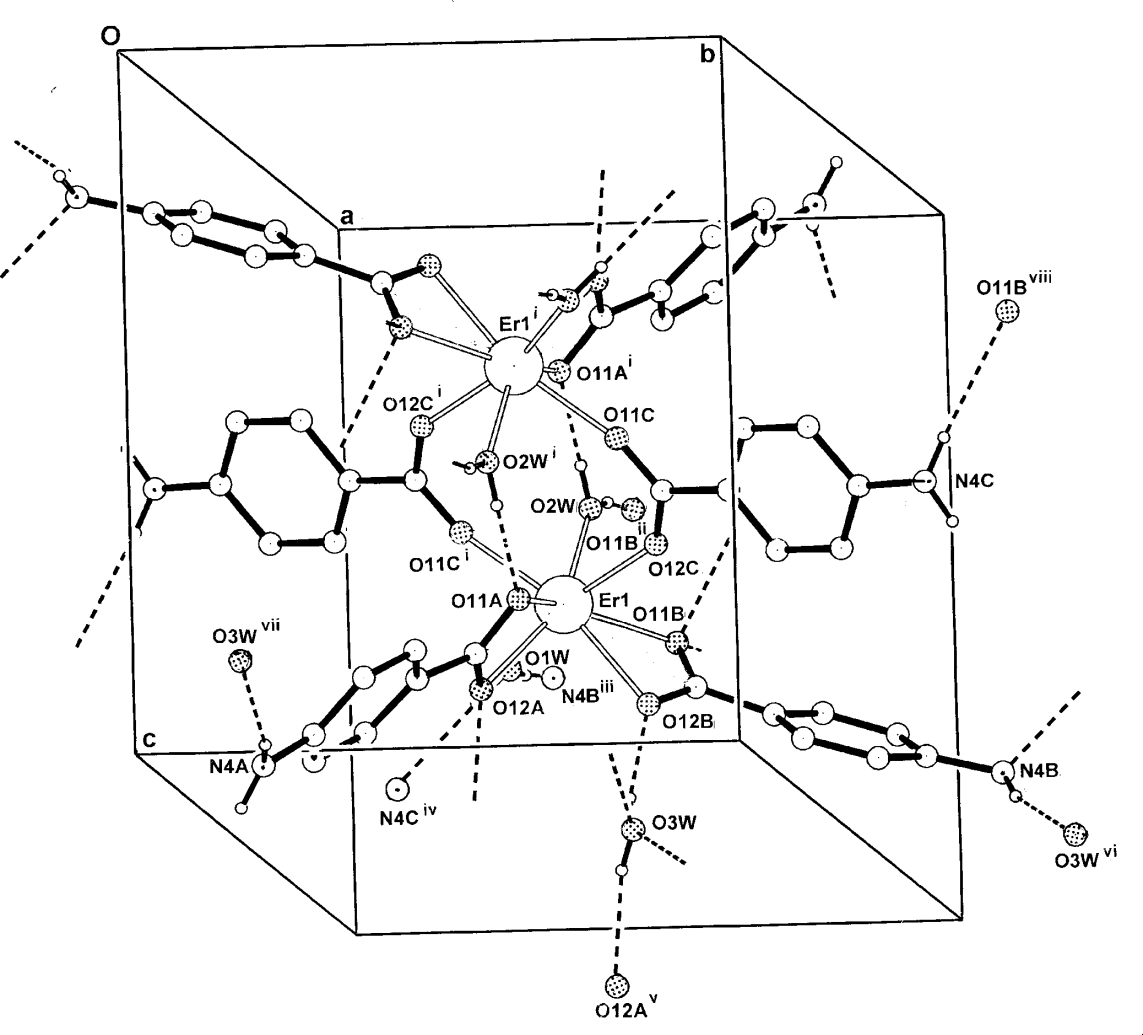
The dimeric complex (I) in the unit cell, viewed approximately down [100], showing intra- and inter­dimer hydrogen-bonding extensions as dashed lines. Non-associative H atoms have been omitted. For symmetry codes, see Table 2 ▸.

In the crystal structure of compound (I), extensive inter-unit O—H⋯O and O—H⋯N hydrogen-bonding inter­actions are present, involving both the coordinating water mol­ecules as well as the solvent water mol­ecules, with carboxyl­ate O-atom acceptors and amine N-atom acceptors (Table 2 ▸). These, together with amine N—H⋯O_water_ and O_carbox­yl_ hydrogen bonds give a three-dimensional network structure (Figs. 4 ▸ and 5 ▸). One H atom of each of the amine groups on the three 4-ABA ligand components of the complex is not involved in hydrogen-bonding. Also present in the supra­molecular structure are weak π–π inter­actions between *A* ligands [ring-centroid separation *A*⋯*A* ^vii^ = 3.711 (3) Å] and *C* ligands [*C*⋯*C* ^viii^ = 3.676 (3) Å] (for symmetry codes, see Table 2 ▸). This dimeric carboxyl­ate-bridged complex mode is similar to that found in the erbium acetate complex [Er_2_(CH_3_CO_2_)_6_(H_2_O)_4_]_2_·6H_2_O (Sawase *et al.*, 1984 ▸).

With (II), present are two weak intra-polymer C—H⋯O hydrogen bonds involving methyl H atoms and both a DMSO O-atom acceptor and a Cl-atom acceptor (Table 4 ▸).

## Synthesis and crystallization   

The title compounds were synthesized by warming together for 10 min, a solution obtained by mixing 5 ml of ethano­lic 4-amino­benzoic acid (1 mmol: 135 mg) [for (I)] or 4-chloro-3-nitro­benzoic acid (1 mmol: 200 mg) [for (II)], with 10 ml of aqueous erbium(III) acetate hexa­hydrate (0.3 mmol: 216 mg). Partial room-temperature evaporation of these solutions provided pale-pink block-like single crystals of (I), suitable for X-ray analysis while a colourless powder was obtained from the preparation of (II). Recrystallization using the slow diffusion of water into a DMSO solution gave minor small crystals of (II), suitable for X-ray analysis.

## Refinement details   

Crystal data, data collection and structure refinements for (I) and (II) are summarized in Table 5 ▸. Hydrogen atoms on all water mol­ecules and the amine groups of the 4-ABA ligands in (I) were located by difference methods and positional parameters were refined with restraints [O—H bond length = 0.85 (2) Å and N—H = 0.88 (2) Å], with *U*_iso_(H) = 1.5*U*_eq_(O) or 1.2*U*_eq_(N). Other H atoms were included in the refinement at calculated positions [C—H(aromatic) = 0.95 Å or C—H(meth­yl) = 0.96 Å, with *U*_iso_(H) = 1.2*U*_eq_(C)(aromatic) or 1.5*U*_eq_(C)(meth­yl)], using a riding-model approximation. In the refinement of (II), a number of large difference electron density residual peaks (5–7 e Å^−3^) located within 1.0 Å of the Er1 site were present. These are possibly due to poor crystal quality coupled to effects of an insufficient absorption correction.

## Supplementary Material

Crystal structure: contains datablock(s) global, I, II. DOI: 10.1107/S2056989015020319/wm5228sup1.cif

Structure factors: contains datablock(s) I. DOI: 10.1107/S2056989015020319/wm5228Isup2.hkl

Structure factors: contains datablock(s) II. DOI: 10.1107/S2056989015020319/wm5228IIsup3.hkl

CCDC references: 1433543, 1433542

Additional supporting information:  crystallographic information; 3D view; checkCIF report

## Figures and Tables

**Figure 5:**
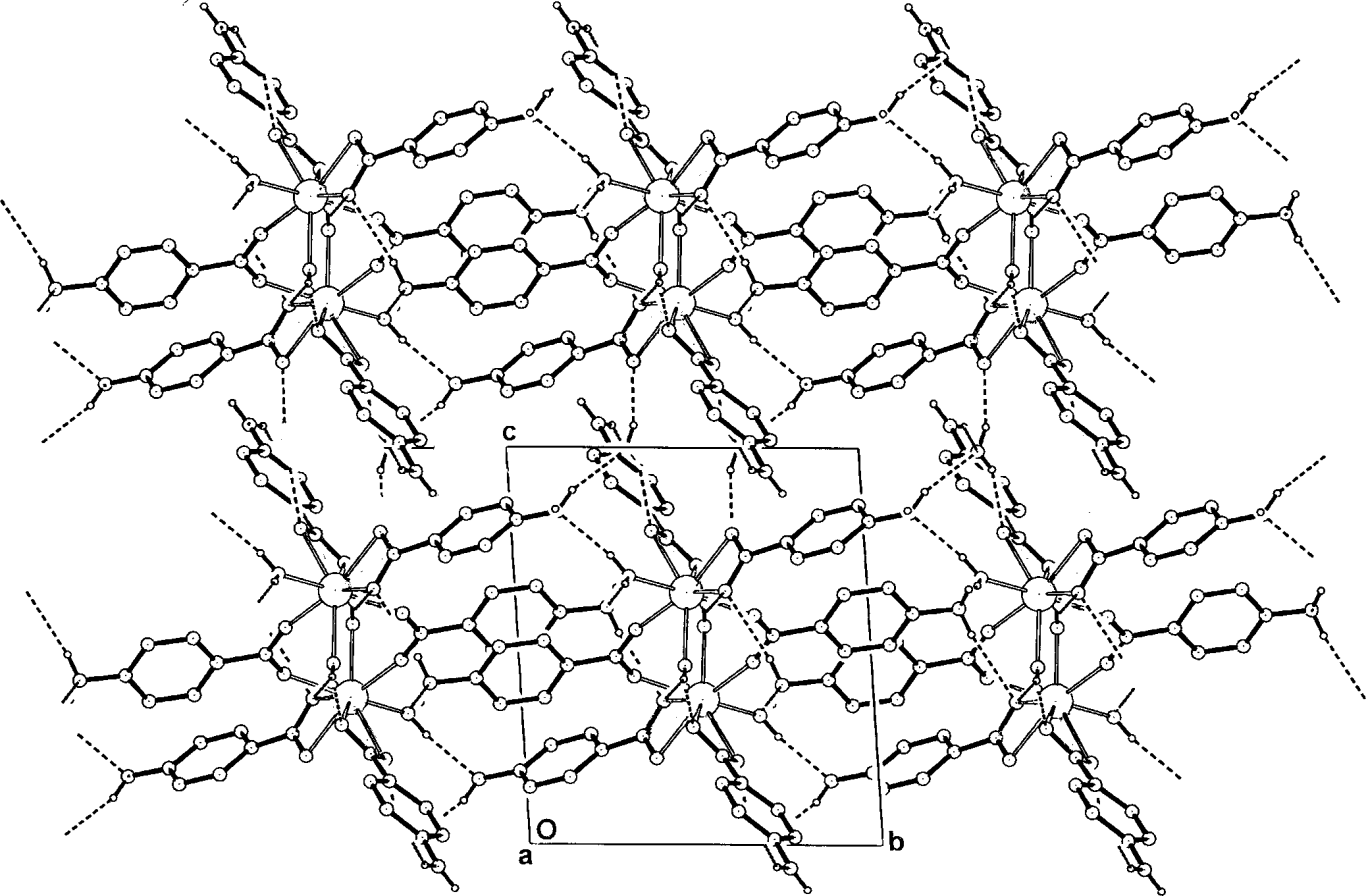
The three-dimensional hydrogen-bonded structure of (I) in the unit cell, viewed along [100]. Non-associative H atoms have been omitted.

**Table 1:** Selected bond lengths (Å) for (I)

Er1—O1*W*	2.373 (2)	Er1—O12*A*	2.333 (3)
Er1—O2*W*	2.295 (3)	Er1—O12*B*	2.385 (3)
Er1—O11*A*	2.477 (3)	Er1—O12*C*	2.232 (3)
Er1—O11*B*	2.478 (3)	Er1—O11*C* ^i^	2.233 (4)

Symmetry code: (i) 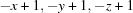.

**Table 2:** Hydrogen-bond geometry (Å, °) for (I)

*D*—H⋯*A*	*D*—H	H⋯*A*	*D*⋯*A*	*D*—H⋯*A*
O1*W*—H11*W*⋯O11*A* ^i^	0.82 (4)	1.95 (4)	2.757 (4)	166 (4)
O1*W*—H12*W*⋯O11*B* ^ii^	0.82 (3)	1.98 (3)	2.777 (4)	163 (4)
O2*W*—H21*W*⋯N4*B* ^iii^	0.84 (4)	2.09 (4)	2.902 (5)	162 (5)
O2*W*—H22*W*⋯N4*C* ^iv^	0.86 (4)	1.89 (4)	2.735 (6)	168 (5)
O3*W*—H31*W*⋯O12*B*	0.83 (4)	1.99 (4)	2.777 (4)	160 (5)
O3*W*—H32*W*⋯O12*A* ^v^	0.85 (5)	2.07 (5)	2.841 (5)	151 (5)
N4*A*—H42*A*⋯O3*W* ^vi^	0.88 (4)	2.08 (4)	2.902 (6)	156 (4)
N4*B*—H41*B*⋯O3*W* ^vii^	0.86 (4)	2.18 (4)	3.014 (6)	164 (4)
N4*C*—H42*C*⋯O11*B* ^viii^	0.86 (3)	2.49 (4)	3.341 (5)	170 (5)

Symmetry codes: (i) 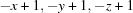; (ii) 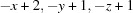; (iii) 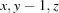; (iv) 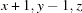; (v) 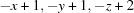; (vi) 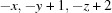; (vii) 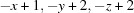; (viii) 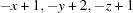.

**Table 3:** Selected bond lengths (Å) for (II)

Er1—O11	2.306 (7)	Er1—O12*C* ^i^	2.287 (6)
Er1—O11*C*	2.312 (8)	Er1—O11*A* ^ii^	2.300 (6)
Er1—O12*A*	2.317 (7)	Er1—O11*B* ^ii^	2.348 (6)
Er1—O12*B*	2.239 (6)		

Symmetry codes: (i) 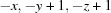; (ii) 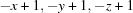.

**Table 4:** Hydrogen-bond geometry (Å, °) for (II)

*D*—H⋯*A*	*D*—H	H⋯*A*	*D*⋯*A*	*D*—H⋯*A*
C2*A*—H2*A*⋯S1	0.95	2.86	3.743 (10)	155
C2*B*—H2*B*⋯O11	0.95	2.56	3.298 (13)	135
C11—H111⋯Cl4*A* ^iii^	0.98	2.79	3.486 (11)	129
C12—H123⋯O32*A* ^iv^	0.98	2.44	3.376 (15)	158

Symmetry codes: (iii) 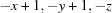; (iv) 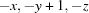.

**Table 5:** Experimental details

	(I)	(II)
Crystal data		
Chemical formula	[Er_2_(C_7_H_6_NO_2_)_6_(H_2_O)_4_]·2H_2_O	[Er_2_(C_7_H_3_ClNO_4_)_6_(C_2_H_6_OS)_2_]
*M*_r_	1259.38	1694.10
Crystal system, space group	Triclinic, *P* 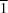	Triclinic, *P* 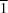
Temperature (K)	200	200
*a*, *b*, *c* (Å)	9.0332 (5), 10.9363 (6), 12.6194 (6)	8.2408 (3), 12.4040 (8), 15.3409 (10)
α, β, γ (°)	89.015 (4), 72.105 (5), 74.814 (5)	111.443 (6), 98.063 (4), 96.684 (4)
*V* (Å^3^)	1142.21 (10)	1421.04 (14)
*Z*	1	1
Radiation type	Mo *K*α	Mo *K*α
μ (mm^−1^)	3.73	3.38
Crystal size (mm)	0.30 × 0.30 × 0.25	0.25 × 0.12 × 0.04

Data collection		
Diffractometer	Oxford Diffraction Gemini-S CCD detector	Oxford Diffraction Gemini-S CCD detector
Absorption correction	Multi-scan (*CrysAlis PRO*; Agilent, 2013 ▸)	Multi-scan (*CrysAlis PRO*; Agilent, 2013 ▸)
*T*_min_, *T*_max_	0.713, 0.980	0.494, 0.980
No. of measured, independent and observed [*I* > 2σ(*I*)] reflections	7274, 4480, 4137	10041, 5566, 4814
*R*_int_	0.035	0.055
(sin θ/λ)_max_ (Å^−1^)	0.617	0.617

Refinement		
*R*[*F* ^2^ > 2σ(*F* ^2^)], *wR*(*F* ^2^), *S*	0.029, 0.058, 1.05	0.067, 0.181, 1.06
No. of reflections	4480	5566
No. of parameters	343	397
No. of restraints	12	0
H-atom treatment	H atoms treated by a mixture of independent and constrained refinement	H-atom parameters constrained
Δρ_max_, Δρ_min_ (e Å^−3^)	1.03, −0.71	6.83, −2.41

Computer programs: *CrysAlis PRO* (Agilent, 2013 ▸), *SIR92* (Altomare *et al.*, 1993 ▸), *SHELXS97* and *SHELXL97* (Sheldrick, 2008 ▸) within *WinGX* (Farrugia, 2012 ▸) and *PLATON* (Spek, 2009 ▸).

## supplementary crystallographic information

### (I) Bis(µ2-4-aminobenzoato-κ2O:O')bis[bis(4-aminobenzoato-κ2O,O')diaquaerbium(III)] dihydrate Crystal data

**Table:**

[Er_2_(C_7_H_6_NO_2_)_6_(H_2_O)_4_]·2H_2_O	*Z* = 1
*M**_r_* = 1259.38	*F*(000) = 622
Triclinic, *P*1	*D*_x_ = 1.831 Mg m^−^^3^
Hall symbol: -P 1	Mo *K*α radiation, λ = 0.71073 Å
*a* = 9.0332 (5) Å	Cell parameters from 3598 reflections
*b* = 10.9363 (6) Å	θ = 3.6–28.8°
*c* = 12.6194 (6) Å	µ = 3.73 mm^−^^1^
α = 89.015 (4)°	*T* = 200 K
β = 72.105 (5)°	Block, pink
γ = 74.814 (5)°	0.30 × 0.30 × 0.25 mm
*V* = 1142.21 (10) Å^3^	

### (I) Bis(µ2-4-aminobenzoato-κ2O:O')bis[bis(4-aminobenzoato-κ2O,O')diaquaerbium(III)] dihydrate Data collection

**Table:**

Oxford Diffraction Gemini-S CCD-detector diffractometer	4480 independent reflections
Radiation source: Enhance (Mo) X-ray source	4137 reflections with *I* > 2σ(*I*)
Graphite monochromator	*R*_int_ = 0.035
Detector resolution: 16.077 pixels mm^-1^	θ_max_ = 26.0°, θ_min_ = 3.4°
ω scans	*h* = −11→11
Absorption correction: multi-scan (CrysAlis PRO; Agilent, 2013)	*k* = −10→13
*T*_min_ = 0.713, *T*_max_ = 0.980	*l* = −15→14
7274 measured reflections	

### (I) Bis(µ2-4-aminobenzoato-κ2O:O')bis[bis(4-aminobenzoato-κ2O,O')diaquaerbium(III)] dihydrate Refinement

**Table:**

Refinement on *F*^2^	Primary atom site location: structure-invariant direct methods
Least-squares matrix: full	Secondary atom site location: difference Fourier map
*R*[*F*^2^ > 2σ(*F*^2^)] = 0.029	Hydrogen site location: inferred from neighbouring sites
*wR*(*F*^2^) = 0.058	H atoms treated by a mixture of independent and constrained refinement
*S* = 1.05	*w* = 1/[σ^2^(*F*_o_^2^) + (0.011*P*)^2^] where *P* = (*F*_o_^2^ + 2*F*_c_^2^)/3
4480 reflections	(Δ/σ)_max_ = 0.002
343 parameters	Δρ_max_ = 1.03 e Å^−^^3^
12 restraints	Δρ_min_ = −0.71 e Å^−^^3^

### (I) Bis(µ2-4-aminobenzoato-κ2O:O')bis[bis(4-aminobenzoato-κ2O,O')diaquaerbium(III)] dihydrate Special details

**Table:**

Geometry. Bond distances, angles *etc*. have been calculated using the rounded fractional coordinates. All su's are estimated from the variances of the (full) variance-covariance matrix. The cell e.s.d.'s are taken into account in the estimation of distances, angles and torsion angles
Refinement. Refinement of *F*^2^ against ALL reflections. The weighted *R*-factor *wR* and goodness of fit *S* are based on *F*^2^, conventional *R*-factors *R* are based on *F*, with *F* set to zero for negative *F*^2^. The threshold expression of *F*^2^ > σ(*F*^2^) is used only for calculating *R*-factors(gt) *etc*. and is not relevant to the choice of reflections for refinement. *R*-factors based on *F*^2^ are statistically about twice as large as those based on *F*, and *R*- factors based on ALL data will be even larger.

### (I) Bis(µ2-4-aminobenzoato-κ2O:O')bis[bis(4-aminobenzoato-κ2O,O')diaquaerbium(III)] dihydrate Fractional atomic coordinates and isotropic or equivalent isotropic displacement parameters (Å^2^)

**Table:**

	*x*	*y*	*z*	*U*_iso_*/*U*_eq_	
Er1	0.63868 (2)	0.48651 (2)	0.63400 (1)	0.0172 (1)	
O1W	0.8216 (3)	0.4689 (3)	0.4504 (2)	0.0236 (9)	
O2W	0.8310 (4)	0.3257 (3)	0.6738 (3)	0.0310 (10)	
O3W	0.4420 (4)	0.6614 (4)	1.0062 (3)	0.0450 (13)	
O11A	0.3424 (3)	0.5182 (3)	0.7029 (2)	0.0229 (9)	
O11B	0.8438 (3)	0.5973 (3)	0.6381 (2)	0.0226 (9)	
O11C	0.4044 (4)	0.6642 (3)	0.4607 (3)	0.0393 (11)	
O12A	0.4967 (3)	0.3977 (3)	0.7885 (2)	0.0279 (10)	
O12B	0.6239 (4)	0.6268 (3)	0.7818 (2)	0.0297 (10)	
O12C	0.5398 (3)	0.6760 (3)	0.5771 (2)	0.0326 (10)	
N4A	−0.1513 (5)	0.2669 (5)	1.0592 (3)	0.0420 (16)	
N4B	0.8254 (5)	1.1355 (4)	0.8371 (3)	0.0338 (14)	
N4C	0.1613 (5)	1.2581 (4)	0.5966 (4)	0.0408 (14)	
C1A	0.2234 (5)	0.3912 (4)	0.8436 (3)	0.0205 (12)	
C1B	0.7719 (5)	0.7812 (4)	0.7614 (3)	0.0209 (11)	
C1C	0.3588 (4)	0.8633 (4)	0.5499 (3)	0.0173 (11)	
C2A	0.2533 (5)	0.2916 (4)	0.9109 (3)	0.0259 (12)	
C2B	0.6425 (5)	0.8743 (4)	0.8302 (3)	0.0245 (12)	
C2C	0.3840 (5)	0.9328 (4)	0.6308 (3)	0.0239 (12)	
C3A	0.1314 (5)	0.2489 (4)	0.9799 (3)	0.0286 (16)	
C3B	0.6601 (5)	0.9903 (4)	0.8557 (3)	0.0269 (12)	
C3C	0.3173 (5)	1.0619 (4)	0.6478 (3)	0.0297 (14)	
C4A	−0.0285 (5)	0.3068 (4)	0.9855 (3)	0.0272 (16)	
C4B	0.8090 (5)	1.0165 (4)	0.8158 (3)	0.0238 (14)	
C4C	0.2265 (5)	1.1253 (4)	0.5836 (4)	0.0264 (14)	
C5A	−0.0601 (5)	0.4036 (4)	0.9147 (3)	0.0284 (14)	
C5B	0.9399 (5)	0.9232 (4)	0.7501 (3)	0.0263 (12)	
C5C	0.1958 (5)	1.0556 (4)	0.5055 (3)	0.0295 (14)	
C6A	0.0656 (5)	0.4452 (4)	0.8453 (3)	0.0240 (12)	
C6B	0.9214 (5)	0.8076 (4)	0.7221 (3)	0.0243 (12)	
C6C	0.2620 (5)	0.9257 (4)	0.4890 (3)	0.0272 (14)	
C11A	0.3588 (5)	0.4394 (4)	0.7749 (3)	0.0205 (12)	
C11B	0.7480 (5)	0.6613 (4)	0.7262 (3)	0.0222 (12)	
C11C	0.4396 (5)	0.7246 (4)	0.5278 (3)	0.0209 (12)	
H2A	0.35910	0.25320	0.90870	0.0310*	
H2B	0.54240	0.85770	0.85940	0.0290*	
H2C	0.44660	0.89160	0.67390	0.0290*	
H3A	0.15460	0.18120	1.02310	0.0350*	
H3B	0.57120	1.05190	0.90000	0.0320*	
H3C	0.33360	1.10700	0.70320	0.0350*	
H5A	−0.16540	0.43970	0.91440	0.0340*	
H5B	1.04110	0.93850	0.72460	0.0310*	
H5C	0.13050	1.09660	0.46410	0.0350*	
H6A	0.04410	0.51030	0.79920	0.0290*	
H6B	1.00980	0.74670	0.67650	0.0290*	
H6C	0.24110	0.87990	0.43650	0.0330*	
H11W	0.788 (5)	0.467 (4)	0.397 (3)	0.0350*	
H12W	0.917 (3)	0.463 (4)	0.417 (3)	0.0350*	
H21W	0.807 (6)	0.278 (4)	0.726 (3)	0.0460*	
H22W	0.934 (3)	0.314 (5)	0.655 (4)	0.0460*	
H41A	−0.136 (6)	0.223 (4)	1.115 (3)	0.0500*	
H41B	0.750 (4)	1.182 (4)	0.891 (3)	0.0400*	
H41C	0.198 (6)	1.289 (5)	0.645 (3)	0.0490*	
H42A	−0.248 (3)	0.305 (4)	1.056 (4)	0.0500*	
H42B	0.919 (3)	1.130 (5)	0.843 (4)	0.0400*	
H42C	0.171 (6)	1.288 (5)	0.532 (2)	0.0490*	
H31W	0.507 (5)	0.634 (5)	0.944 (3)	0.0680*	
H32W	0.489 (6)	0.624 (5)	1.051 (4)	0.0680*	

### (I) Bis(µ2-4-aminobenzoato-κ2O:O')bis[bis(4-aminobenzoato-κ2O,O')diaquaerbium(III)] dihydrate Atomic displacement parameters (Å^2^)

**Table:**

	*U*^11^	*U*^22^	*U*^33^	*U*^12^	*U*^13^	*U*^23^
Er1	0.0190 (1)	0.0146 (1)	0.0170 (1)	−0.0037 (1)	−0.0048 (1)	0.0002 (1)
O1W	0.0247 (16)	0.0303 (17)	0.0135 (14)	−0.0055 (14)	−0.0043 (12)	−0.0005 (13)
O2W	0.0219 (16)	0.0321 (19)	0.0371 (19)	−0.0049 (15)	−0.0093 (15)	0.0164 (15)
O11A	0.0254 (15)	0.0245 (16)	0.0175 (14)	−0.0052 (13)	−0.0063 (12)	0.0069 (12)
O11B	0.0220 (15)	0.0253 (16)	0.0201 (14)	−0.0065 (13)	−0.0057 (12)	−0.0061 (12)
O11C	0.044 (2)	0.0294 (19)	0.0392 (19)	−0.0148 (16)	−0.0004 (16)	−0.0138 (15)
O12A	0.0244 (16)	0.0355 (18)	0.0277 (16)	−0.0105 (14)	−0.0123 (13)	0.0103 (14)
O12B	0.0364 (18)	0.0329 (18)	0.0211 (15)	−0.0207 (15)	−0.0009 (13)	−0.0038 (13)
O12C	0.0249 (17)	0.0214 (17)	0.047 (2)	−0.0014 (13)	−0.0095 (15)	0.0120 (15)
N4A	0.042 (3)	0.062 (3)	0.030 (2)	−0.030 (3)	−0.010 (2)	0.015 (2)
N4B	0.049 (3)	0.022 (2)	0.030 (2)	−0.013 (2)	−0.009 (2)	−0.0008 (17)
N4C	0.028 (2)	0.020 (2)	0.061 (3)	−0.0033 (18)	0.002 (2)	0.005 (2)
C1A	0.024 (2)	0.020 (2)	0.017 (2)	−0.0060 (18)	−0.0057 (17)	−0.0019 (17)
C1B	0.028 (2)	0.021 (2)	0.0153 (19)	−0.0092 (19)	−0.0069 (17)	0.0001 (17)
C1C	0.0153 (19)	0.016 (2)	0.019 (2)	−0.0049 (16)	−0.0023 (16)	−0.0001 (16)
C2A	0.024 (2)	0.028 (2)	0.028 (2)	−0.0071 (19)	−0.0114 (19)	0.0045 (19)
C2B	0.025 (2)	0.025 (2)	0.021 (2)	−0.0074 (19)	−0.0033 (18)	0.0024 (18)
C2C	0.026 (2)	0.022 (2)	0.026 (2)	−0.0041 (19)	−0.0133 (18)	−0.0007 (18)
C3A	0.036 (3)	0.028 (3)	0.027 (2)	−0.013 (2)	−0.014 (2)	0.011 (2)
C3B	0.031 (2)	0.021 (2)	0.022 (2)	−0.0007 (19)	−0.0041 (18)	−0.0032 (18)
C3C	0.033 (3)	0.024 (2)	0.032 (2)	−0.007 (2)	−0.010 (2)	−0.010 (2)
C4A	0.034 (3)	0.034 (3)	0.020 (2)	−0.021 (2)	−0.0077 (19)	0.0017 (19)
C4B	0.040 (3)	0.016 (2)	0.015 (2)	−0.0080 (19)	−0.0080 (18)	0.0036 (17)
C4C	0.020 (2)	0.013 (2)	0.038 (3)	−0.0043 (18)	0.0024 (19)	0.0033 (19)
C5A	0.019 (2)	0.045 (3)	0.023 (2)	−0.012 (2)	−0.0061 (18)	−0.004 (2)
C5B	0.032 (2)	0.026 (2)	0.022 (2)	−0.013 (2)	−0.0057 (19)	0.0028 (19)
C5C	0.030 (2)	0.027 (3)	0.029 (2)	−0.001 (2)	−0.012 (2)	0.011 (2)
C6A	0.028 (2)	0.026 (2)	0.018 (2)	−0.0065 (19)	−0.0082 (18)	0.0033 (18)
C6B	0.031 (2)	0.018 (2)	0.021 (2)	−0.0050 (19)	−0.0052 (18)	0.0007 (17)
C6C	0.030 (2)	0.032 (3)	0.022 (2)	−0.007 (2)	−0.0127 (19)	0.0006 (19)
C11A	0.026 (2)	0.020 (2)	0.018 (2)	−0.0092 (18)	−0.0077 (17)	0.0001 (17)
C11B	0.027 (2)	0.024 (2)	0.021 (2)	−0.0107 (19)	−0.0117 (18)	0.0001 (18)
C11C	0.019 (2)	0.019 (2)	0.021 (2)	−0.0110 (18)	0.0039 (17)	0.0000 (17)
O3W	0.031 (2)	0.071 (3)	0.0254 (18)	−0.0003 (19)	−0.0093 (15)	0.0010 (18)

### (I) Bis(µ2-4-aminobenzoato-κ2O:O')bis[bis(4-aminobenzoato-κ2O,O')diaquaerbium(III)] dihydrate Geometric parameters (Å, º)

**Table:**

Er1—O1W	2.373 (2)	C1A—C2A	1.391 (6)
Er1—O2W	2.295 (3)	C1B—C2B	1.393 (6)
Er1—O11A	2.477 (3)	C1B—C6B	1.393 (7)
Er1—O11B	2.478 (3)	C1B—C11B	1.480 (6)
Er1—O12A	2.333 (3)	C1C—C11C	1.490 (6)
Er1—O12B	2.385 (3)	C1C—C6C	1.380 (6)
Er1—O12C	2.232 (3)	C1C—C2C	1.390 (6)
Er1—O11C^i^	2.233 (4)	C2A—C3A	1.362 (6)
O11A—C11A	1.257 (5)	C2B—C3B	1.375 (6)
O11B—C11B	1.262 (5)	C2C—C3C	1.374 (6)
O11C—C11C	1.245 (6)	C3A—C4A	1.397 (7)
O12A—C11A	1.273 (6)	C3B—C4B	1.388 (7)
O12B—C11B	1.273 (6)	C3C—C4C	1.379 (6)
O12C—C11C	1.254 (5)	C4A—C5A	1.402 (6)
O1W—H12W	0.82 (3)	C4B—C5B	1.386 (6)
O1W—H11W	0.82 (4)	C4C—C5C	1.391 (6)
O2W—H21W	0.84 (4)	C5A—C6A	1.382 (6)
O2W—H22W	0.86 (4)	C5B—C6B	1.383 (6)
O3W—H31W	0.83 (4)	C5C—C6C	1.381 (6)
O3W—H32W	0.85 (5)	C2A—H2A	0.9300
N4A—C4A	1.375 (6)	C2B—H2B	0.9300
N4B—C4B	1.388 (6)	C2C—H2C	0.9300
N4C—C4C	1.409 (6)	C3A—H3A	0.9300
N4A—H41A	0.87 (4)	C3B—H3B	0.9300
N4A—H42A	0.88 (4)	C3C—H3C	0.9300
N4B—H41B	0.86 (4)	C5A—H5A	0.9300
N4B—H42B	0.86 (3)	C5B—H5B	0.9300
N4C—H41C	0.89 (5)	C5C—H5C	0.9300
N4C—H42C	0.86 (3)	C6A—H6A	0.9300
C1A—C11A	1.482 (6)	C6B—H6B	0.9300
C1A—C6A	1.386 (7)	C6C—H6C	0.9300
			
O1W—Er1—O2W	87.02 (12)	C2C—C1C—C6C	118.8 (4)
O1W—Er1—O11A	131.43 (9)	C6C—C1C—C11C	121.1 (4)
O1W—Er1—O11B	72.20 (9)	C1A—C2A—C3A	121.6 (4)
O1W—Er1—O12A	151.62 (11)	C1B—C2B—C3B	121.2 (4)
O1W—Er1—O12B	124.33 (11)	C1C—C2C—C3C	120.6 (4)
O1W—Er1—O12C	79.98 (10)	C2A—C3A—C4A	119.9 (4)
O1W—Er1—O11C^i^	73.68 (12)	C2B—C3B—C4B	120.7 (4)
O2W—Er1—O11A	126.78 (12)	C2C—C3C—C4C	120.6 (4)
O2W—Er1—O11B	78.50 (12)	C3A—C4A—C5A	119.1 (4)
O2W—Er1—O12A	75.02 (12)	N4A—C4A—C5A	121.4 (4)
O2W—Er1—O12B	93.16 (12)	N4A—C4A—C3A	119.5 (4)
O2W—Er1—O12C	156.11 (12)	C3B—C4B—C5B	118.7 (4)
O2W—Er1—O11C^i^	85.80 (13)	N4B—C4B—C5B	120.5 (4)
O11A—Er1—O11B	140.04 (10)	N4B—C4B—C3B	120.8 (4)
O11A—Er1—O12A	53.86 (10)	C3C—C4C—C5C	118.9 (4)
O11A—Er1—O12B	91.09 (11)	N4C—C4C—C3C	121.9 (4)
O11A—Er1—O12C	76.09 (10)	N4C—C4C—C5C	119.2 (4)
O11A—Er1—O11C^i^	75.35 (12)	C4A—C5A—C6A	119.8 (4)
O11B—Er1—O12A	123.63 (9)	C4B—C5B—C6B	120.6 (4)
O11B—Er1—O12B	53.56 (10)	C4C—C5C—C6C	120.3 (4)
O11B—Er1—O12C	78.48 (10)	C1A—C6A—C5A	120.8 (4)
O11B—Er1—O11C^i^	142.95 (11)	C1B—C6B—C5B	120.8 (4)
O12A—Er1—O12B	79.21 (10)	C1C—C6C—C5C	120.6 (4)
O12A—Er1—O12C	123.94 (10)	O11A—C11A—C1A	122.2 (4)
O11C^i^—Er1—O12A	83.11 (12)	O12A—C11A—C1A	118.5 (4)
O12B—Er1—O12C	78.15 (10)	O11A—C11A—O12A	119.2 (4)
O11C^i^—Er1—O12B	161.93 (12)	O11B—C11B—C1B	120.7 (4)
O11C^i^—Er1—O12C	109.26 (11)	O12B—C11B—C1B	119.4 (3)
Er1—O11A—C11A	90.0 (3)	O11B—C11B—O12B	119.8 (4)
Er1—O11B—C11B	90.2 (3)	O11C—C11C—O12C	124.0 (4)
Er1^i^—O11C—C11C	165.0 (3)	O11C—C11C—C1C	117.9 (4)
Er1—O12A—C11A	96.3 (2)	O12C—C11C—C1C	118.1 (4)
Er1—O12B—C11B	94.2 (2)	C1A—C2A—H2A	119.00
Er1—O12C—C11C	138.1 (3)	C3A—C2A—H2A	119.00
H11W—O1W—H12W	100 (4)	C3B—C2B—H2B	119.00
Er1—O1W—H11W	119 (3)	C1B—C2B—H2B	119.00
Er1—O1W—H12W	141 (2)	C1C—C2C—H2C	120.00
H21W—O2W—H22W	107 (5)	C3C—C2C—H2C	120.00
Er1—O2W—H21W	122 (4)	C4A—C3A—H3A	120.00
Er1—O2W—H22W	130 (3)	C2A—C3A—H3A	120.00
H31W—O3W—H32W	104 (5)	C2B—C3B—H3B	120.00
C4A—N4A—H41A	121 (4)	C4B—C3B—H3B	120.00
H41A—N4A—H42A	122 (5)	C4C—C3C—H3C	120.00
C4A—N4A—H42A	115 (3)	C2C—C3C—H3C	120.00
C4B—N4B—H42B	111 (4)	C4A—C5A—H5A	120.00
H41B—N4B—H42B	112 (4)	C6A—C5A—H5A	120.00
C4B—N4B—H41B	116 (3)	C6B—C5B—H5B	120.00
C4C—N4C—H41C	108 (3)	C4B—C5B—H5B	120.00
H41C—N4C—H42C	121 (5)	C4C—C5C—H5C	120.00
C4C—N4C—H42C	110 (3)	C6C—C5C—H5C	120.00
C2A—C1A—C6A	118.6 (4)	C5A—C6A—H6A	120.00
C6A—C1A—C11A	121.7 (4)	C1A—C6A—H6A	120.00
C2A—C1A—C11A	119.7 (4)	C1B—C6B—H6B	120.00
C2B—C1B—C11B	120.6 (4)	C5B—C6B—H6B	120.00
C6B—C1B—C11B	121.3 (4)	C1C—C6C—H6C	120.00
C2B—C1B—C6B	118.0 (4)	C5C—C6C—H6C	120.00
C2C—C1C—C11C	120.1 (4)		
			
O1W—Er1—O11A—C11A	139.1 (2)	Er1—O12C—C11C—C1C	153.5 (3)
O2W—Er1—O11A—C11A	14.1 (3)	C2A—C1A—C6A—C5A	1.9 (6)
O11B—Er1—O11A—C11A	−106.2 (3)	C6A—C1A—C2A—C3A	−1.8 (6)
O12A—Er1—O11A—C11A	−4.9 (2)	C11A—C1A—C2A—C3A	176.4 (4)
O12B—Er1—O11A—C11A	−80.7 (2)	C6A—C1A—C11A—O11A	−10.6 (6)
O12C—Er1—O11A—C11A	−158.2 (2)	C6A—C1A—C11A—O12A	170.5 (4)
O11C^i^—Er1—O11A—C11A	87.3 (2)	C11A—C1A—C6A—C5A	−176.3 (4)
O1W—Er1—O11B—C11B	158.2 (3)	C2A—C1A—C11A—O11A	171.2 (4)
O2W—Er1—O11B—C11B	−111.2 (2)	C2A—C1A—C11A—O12A	−7.7 (6)
O11A—Er1—O11B—C11B	23.9 (3)	C6B—C1B—C2B—C3B	2.1 (6)
O12A—Er1—O11B—C11B	−48.1 (3)	C11B—C1B—C2B—C3B	−174.4 (4)
O12B—Er1—O11B—C11B	−8.5 (2)	C2B—C1B—C6B—C5B	−0.5 (6)
O12C—Er1—O11B—C11B	75.2 (2)	C2B—C1B—C11B—O11B	155.9 (4)
O11C^i^—Er1—O11B—C11B	−178.1 (2)	C2B—C1B—C11B—O12B	−19.8 (6)
O1W—Er1—O12A—C11A	−107.0 (3)	C6B—C1B—C11B—O11B	−20.5 (6)
O2W—Er1—O12A—C11A	−159.5 (3)	C6B—C1B—C11B—O12B	163.9 (4)
O11A—Er1—O12A—C11A	4.9 (2)	C11B—C1B—C6B—C5B	176.0 (4)
O11B—Er1—O12A—C11A	135.7 (2)	C6C—C1C—C2C—C3C	−1.7 (6)
O12B—Er1—O12A—C11A	104.2 (3)	C11C—C1C—C2C—C3C	176.5 (4)
O12C—Er1—O12A—C11A	36.6 (3)	C2C—C1C—C11C—O12C	−5.8 (6)
O11C^i^—Er1—O12A—C11A	−72.0 (2)	C6C—C1C—C11C—O11C	−7.1 (6)
O1W—Er1—O12B—C11B	−6.9 (3)	C6C—C1C—C11C—O12C	172.3 (4)
O2W—Er1—O12B—C11B	81.7 (3)	C2C—C1C—C6C—C5C	2.2 (6)
O11A—Er1—O12B—C11B	−151.4 (3)	C11C—C1C—C6C—C5C	−176.0 (4)
O11B—Er1—O12B—C11B	8.5 (2)	C2C—C1C—C11C—O11C	174.8 (4)
O12A—Er1—O12B—C11B	155.8 (3)	C1A—C2A—C3A—C4A	−1.0 (6)
O12C—Er1—O12B—C11B	−75.9 (3)	C1B—C2B—C3B—C4B	−1.8 (6)
O1W—Er1—O12C—C11C	88.7 (4)	C1C—C2C—C3C—C4C	−1.2 (7)
O2W—Er1—O12C—C11C	146.8 (4)	C2A—C3A—C4A—C5A	3.7 (6)
O11A—Er1—O12C—C11C	−48.7 (4)	C2A—C3A—C4A—N4A	−177.0 (4)
O11B—Er1—O12C—C11C	162.4 (4)	C2B—C3B—C4B—C5B	−0.3 (6)
O12A—Er1—O12C—C11C	−74.6 (4)	C2B—C3B—C4B—N4B	177.0 (4)
O12B—Er1—O12C—C11C	−142.8 (4)	C2C—C3C—C4C—N4C	−177.8 (4)
O11C^i^—Er1—O12C—C11C	20.1 (4)	C2C—C3C—C4C—C5C	3.6 (7)
Er1—O11A—C11A—O12A	8.3 (4)	N4A—C4A—C5A—C6A	177.1 (4)
Er1—O11A—C11A—C1A	−170.6 (3)	C3A—C4A—C5A—C6A	−3.7 (6)
Er1—O11B—C11B—O12B	14.9 (4)	C3B—C4B—C5B—C6B	1.9 (6)
Er1—O11B—C11B—C1B	−160.7 (4)	N4B—C4B—C5B—C6B	−175.4 (4)
Er1—O12A—C11A—O11A	−8.9 (4)	C3C—C4C—C5C—C6C	−3.1 (7)
Er1—O12A—C11A—C1A	170.1 (3)	N4C—C4C—C5C—C6C	178.2 (4)
Er1—O12B—C11B—O11B	−15.5 (4)	C4A—C5A—C6A—C1A	0.9 (6)
Er1—O12B—C11B—C1B	160.1 (3)	C4B—C5B—C6B—C1B	−1.5 (6)
Er1—O12C—C11C—O11C	−27.1 (6)	C4C—C5C—C6C—C1C	0.2 (7)

Symmetry code: (i) −*x*+1, −*y*+1, −*z*+1.

### (I) Bis(µ2-4-aminobenzoato-κ2O:O')bis[bis(4-aminobenzoato-κ2O,O')diaquaerbium(III)] dihydrate Hydrogen-bond geometry (Å, º)

**Table:**

*D*—H···*A*	*D*—H	H···*A*	*D*···*A*	*D*—H···*A*
O1*W*—H11*W*···O11*A*^i^	0.82 (4)	1.95 (4)	2.757 (4)	166 (4)
O1*W*—H12*W*···O11*B*^ii^	0.82 (3)	1.98 (3)	2.777 (4)	163 (4)
O2*W*—H21*W*···N4*B*^iii^	0.84 (4)	2.09 (4)	2.902 (5)	162 (5)
O2*W*—H22*W*···N4*C*^iv^	0.86 (4)	1.89 (4)	2.735 (6)	168 (5)
O3*W*—H31*W*···O12*B*	0.83 (4)	1.99 (4)	2.777 (4)	160 (5)
O3*W*—H32*W*···O12*A*^v^	0.85 (5)	2.07 (5)	2.841 (5)	151 (5)
N4*A*—H42*A*···O3*W*^vi^	0.88 (4)	2.08 (4)	2.902 (6)	156 (4)
N4*B*—H41*B*···O3*W*^vii^	0.86 (4)	2.18 (4)	3.014 (6)	164 (4)
N4*C*—H42*C*···O11*B*^viii^	0.86 (3)	2.49 (4)	3.341 (5)	170 (5)

Symmetry codes: (i) −*x*+1, −*y*+1, −*z*+1; (ii) −*x*+2, −*y*+1, −*z*+1; (iii) *x*, *y*−1, *z*; (iv) *x*+1, *y*−1, *z*; (v) −*x*+1, −*y*+1, −*z*+2; (vi) −*x*, −*y*+1, −*z*+2; (vii) −*x*+1, −*y*+2, −*z*+2; (viii) −*x*+1, −*y*+2, −*z*+1.

### (II) Poly[hexakis(µ2-4-chloro-3-nitrobenzoato-κ2O:O')bis(dimethyl sulfoxide-κO)dierbium(III)] Crystal data

**Table:**

[Er_2_(C_7_H_3_ClNO_4_)_6_(C_2_H_6_OS)_2_]	*Z* = 1
*M**_r_* = 1694.10	*F*(000) = 826
Triclinic, *P*1	*D*_x_ = 1.980 Mg m^−^^3^
Hall symbol: -P 1	Mo *K*α radiation, λ = 0.71073 Å
*a* = 8.2408 (3) Å	Cell parameters from 4326 reflections
*b* = 12.4040 (8) Å	θ = 3.6–28.8°
*c* = 15.3409 (10) Å	µ = 3.38 mm^−^^1^
α = 111.443 (6)°	*T* = 200 K
β = 98.063 (4)°	Prism, colourless
γ = 96.684 (4)°	0.25 × 0.12 × 0.04 mm
*V* = 1421.04 (14) Å^3^	

### (II) Poly[hexakis(µ2-4-chloro-3-nitrobenzoato-κ2O:O')bis(dimethyl sulfoxide-κO)dierbium(III)] Data collection

**Table:**

Oxford Diffraction Gemini-S CCD-detector diffractometer	5566 independent reflections
Radiation source: fine-focus sealed tube	4814 reflections with *I* > 2σ(*I*)
Graphite monochromator	*R*_int_ = 0.055
Detector resolution: 16.077 pixels mm^-1^	θ_max_ = 26.0°, θ_min_ = 3.1°
ω scans	*h* = −10→10
Absorption correction: multi-scan (CrysAlis PRO; Agilent, 2013)	*k* = −15→13
*T*_min_ = 0.494, *T*_max_ = 0.980	*l* = −16→18
10041 measured reflections	

### (II) Poly[hexakis(µ2-4-chloro-3-nitrobenzoato-κ2O:O')bis(dimethyl sulfoxide-κO)dierbium(III)] Refinement

**Table:**

Refinement on *F*^2^	Primary atom site location: structure-invariant direct methods
Least-squares matrix: full	Secondary atom site location: difference Fourier map
*R*[*F*^2^ > 2σ(*F*^2^)] = 0.067	Hydrogen site location: inferred from neighbouring sites
*wR*(*F*^2^) = 0.181	H-atom parameters constrained
*S* = 1.06	*w* = 1/[σ^2^(*F*_o_^2^) + (0.1243*P*)^2^] where *P* = (*F*_o_^2^ + 2*F*_c_^2^)/3
5566 reflections	(Δ/σ)_max_ = 0.001
397 parameters	Δρ_max_ = 6.83 e Å^−^^3^
0 restraints	Δρ_min_ = −2.41 e Å^−^^3^

### (II) Poly[hexakis(µ2-4-chloro-3-nitrobenzoato-κ2O:O')bis(dimethyl sulfoxide-κO)dierbium(III)] Special details

**Table:**

Geometry. Bond distances, angles *etc*. have been calculated using the rounded fractional coordinates. All su's are estimated from the variances of the (full) variance-covariance matrix. The cell e.s.d.'s are taken into account in the estimation of distances, angles and torsion angles
Refinement. Refinement of *F*^2^ against ALL reflections. The weighted *R*-factor *wR* and goodness of fit *S* are based on *F*^2^, conventional *R*-factors *R* are based on *F*, with *F* set to zero for negative *F*^2^. The threshold expression of *F*^2^ > σ(*F*^2^) is used only for calculating *R*-factors(gt) *etc*. and is not relevant to the choice of reflections for refinement. *R*-factors based on *F*^2^ are statistically about twice as large as those based on *F*, and *R*- factors based on ALL data will be even larger.

### (II) Poly[hexakis(µ2-4-chloro-3-nitrobenzoato-κ2O:O')bis(dimethyl sulfoxide-κO)dierbium(III)] Fractional atomic coordinates and isotropic or equivalent isotropic displacement parameters (Å^2^)

**Table:**

	*x*	*y*	*z*	*U*_iso_*/*U*_eq_	
Er1	0.24949 (4)	0.48443 (3)	0.46092 (2)	0.0175 (1)	
Cl4A	0.6408 (5)	0.7116 (4)	0.0335 (3)	0.0699 (16)	
Cl4B	0.2887 (4)	−0.1334 (3)	0.0200 (2)	0.0627 (10)	
Cl4C	−0.3399 (4)	−0.1283 (2)	0.5158 (2)	0.0452 (9)	
S1	0.0342 (3)	0.4386 (2)	0.23184 (16)	0.0269 (7)	
O11	0.1349 (8)	0.3972 (6)	0.2999 (5)	0.0294 (19)	
O11A	0.6659 (7)	0.5633 (6)	0.4105 (4)	0.0250 (19)	
O11B	0.6883 (7)	0.3352 (5)	0.4066 (4)	0.0256 (17)	
O11C	0.0768 (7)	0.3102 (6)	0.4347 (5)	0.027 (2)	
O12A	0.3978 (7)	0.5899 (6)	0.3912 (4)	0.0259 (17)	
O12B	0.4342 (7)	0.3679 (5)	0.4117 (4)	0.0239 (17)	
O12C	−0.0361 (7)	0.4170 (5)	0.5538 (5)	0.0231 (19)	
O31A	0.1634 (13)	0.6185 (12)	0.0929 (8)	0.079 (5)	
O31B	−0.0284 (11)	0.0741 (13)	0.1852 (10)	0.128 (6)	
O31C	−0.1757 (16)	0.1537 (14)	0.7463 (8)	0.112 (6)	
O32A	0.3085 (15)	0.5798 (10)	−0.0175 (7)	0.075 (4)	
O32B	−0.0018 (15)	−0.0583 (16)	0.0725 (12)	0.174 (7)	
O32C	−0.4244 (12)	0.0843 (11)	0.6745 (8)	0.074 (4)	
N3A	0.2942 (15)	0.6108 (9)	0.0664 (7)	0.050 (4)	
N3B	0.0575 (12)	0.0190 (9)	0.1417 (8)	0.050 (3)	
N3C	−0.2816 (13)	0.1149 (8)	0.6759 (7)	0.043 (3)	
C1A	0.5617 (11)	0.6222 (8)	0.2856 (6)	0.023 (2)	
C1B	0.4672 (11)	0.1949 (8)	0.2879 (6)	0.023 (3)	
C1C	−0.0974 (10)	0.2075 (8)	0.5005 (6)	0.023 (3)	
C2A	0.4248 (11)	0.6144 (8)	0.2190 (6)	0.025 (3)	
C2B	0.2996 (11)	0.1571 (9)	0.2529 (7)	0.029 (3)	
C2C	−0.1560 (10)	0.2099 (8)	0.5823 (7)	0.024 (3)	
C3A	0.4480 (14)	0.6353 (9)	0.1384 (7)	0.036 (3)	
C3B	0.2401 (12)	0.0547 (9)	0.1717 (7)	0.033 (3)	
C3C	−0.2293 (12)	0.1085 (9)	0.5859 (7)	0.031 (3)	
C4A	0.6027 (15)	0.6725 (10)	0.1270 (8)	0.038 (3)	
C4B	0.3490 (13)	−0.0081 (9)	0.1221 (7)	0.036 (3)	
C4C	−0.2491 (11)	0.0009 (8)	0.5095 (8)	0.029 (3)	
C5A	0.7399 (13)	0.6842 (10)	0.1966 (8)	0.038 (3)	
C5B	0.5197 (13)	0.0283 (9)	0.1574 (8)	0.036 (3)	
C5C	−0.1928 (13)	−0.0026 (8)	0.4290 (8)	0.034 (3)	
C6A	0.7202 (11)	0.6582 (9)	0.2742 (8)	0.033 (3)	
C6B	0.5809 (12)	0.1291 (8)	0.2402 (7)	0.028 (3)	
C6C	−0.1147 (12)	0.1003 (8)	0.4245 (7)	0.026 (3)	
C11	0.0742 (13)	0.3526 (11)	0.1178 (7)	0.041 (4)	
C11A	0.5391 (10)	0.5897 (7)	0.3704 (6)	0.018 (2)	
C11B	0.5342 (10)	0.3057 (7)	0.3743 (6)	0.018 (3)	
C11C	−0.0137 (10)	0.3191 (8)	0.4954 (6)	0.021 (3)	
C12	−0.1761 (12)	0.3732 (10)	0.2168 (7)	0.035 (3)	
H2A	0.31540	0.59490	0.22840	0.0300*	
H2B	0.22320	0.20130	0.28450	0.0350*	
H2C	−0.14440	0.28250	0.63530	0.0280*	
H5A	0.84870	0.71060	0.18990	0.0460*	
H5B	0.59520	−0.01580	0.12480	0.0430*	
H5C	−0.20710	−0.07540	0.37600	0.0400*	
H6A	0.81490	0.66480	0.32030	0.0400*	
H6B	0.69750	0.15340	0.26440	0.0340*	
H6C	−0.07290	0.09710	0.36910	0.0310*	
H111	0.18920	0.37880	0.11450	0.0610*	
H112	−0.00280	0.36250	0.06760	0.0610*	
H113	0.05800	0.26940	0.10880	0.0610*	
H121	−0.21770	0.41140	0.27530	0.0530*	
H122	−0.18320	0.28910	0.20350	0.0530*	
H123	−0.24380	0.38300	0.16330	0.0530*	

### (II) Poly[hexakis(µ2-4-chloro-3-nitrobenzoato-κ2O:O')bis(dimethyl sulfoxide-κO)dierbium(III)] Atomic displacement parameters (Å^2^)

**Table:**

	*U*^11^	*U*^22^	*U*^33^	*U*^12^	*U*^13^	*U*^23^
Er1	0.0131 (2)	0.0219 (2)	0.0184 (2)	0.0064 (2)	0.0031 (2)	0.0079 (2)
Cl4A	0.092 (3)	0.105 (3)	0.061 (2)	0.055 (2)	0.052 (2)	0.063 (2)
Cl4B	0.0548 (18)	0.0484 (17)	0.0474 (18)	0.0099 (14)	−0.0065 (14)	−0.0183 (14)
Cl4C	0.0525 (17)	0.0285 (13)	0.0578 (18)	−0.0038 (12)	0.0126 (13)	0.0231 (12)
S1	0.0227 (11)	0.0357 (13)	0.0211 (11)	0.0059 (9)	0.0017 (8)	0.0105 (9)
O11	0.031 (3)	0.031 (3)	0.026 (4)	0.016 (3)	0.002 (3)	0.009 (3)
O11A	0.017 (3)	0.035 (4)	0.025 (3)	0.010 (3)	0.005 (2)	0.012 (3)
O11B	0.016 (3)	0.033 (3)	0.028 (3)	0.005 (3)	0.004 (2)	0.012 (3)
O11C	0.016 (3)	0.035 (4)	0.028 (4)	0.000 (3)	0.005 (3)	0.012 (3)
O12A	0.021 (3)	0.035 (3)	0.028 (3)	0.008 (3)	0.006 (3)	0.018 (3)
O12B	0.020 (3)	0.026 (3)	0.027 (3)	0.010 (3)	0.008 (2)	0.009 (3)
O12C	0.015 (3)	0.017 (3)	0.034 (4)	0.005 (2)	0.001 (2)	0.007 (3)
O31A	0.048 (6)	0.135 (10)	0.064 (7)	0.021 (6)	−0.010 (5)	0.057 (7)
O31B	0.017 (4)	0.158 (13)	0.111 (10)	−0.001 (6)	0.005 (5)	−0.052 (9)
O31C	0.094 (9)	0.168 (13)	0.041 (6)	−0.061 (9)	−0.002 (6)	0.035 (7)
O32A	0.106 (8)	0.081 (7)	0.034 (5)	0.026 (6)	−0.011 (5)	0.025 (5)
O32B	0.039 (6)	0.176 (15)	0.154 (14)	0.001 (8)	−0.016 (7)	−0.092 (12)
O32C	0.051 (6)	0.119 (9)	0.081 (7)	0.017 (6)	0.039 (5)	0.064 (7)
N3A	0.060 (7)	0.058 (6)	0.033 (6)	0.020 (5)	−0.009 (5)	0.022 (5)
N3B	0.032 (5)	0.055 (6)	0.046 (6)	0.008 (5)	−0.006 (4)	0.004 (5)
N3C	0.059 (6)	0.035 (5)	0.034 (5)	−0.001 (4)	0.015 (5)	0.014 (4)
C1A	0.022 (4)	0.027 (4)	0.020 (4)	0.005 (4)	0.005 (3)	0.010 (4)
C1B	0.017 (4)	0.027 (4)	0.023 (5)	0.005 (3)	0.002 (3)	0.007 (4)
C1C	0.017 (4)	0.026 (4)	0.025 (5)	0.004 (3)	0.005 (3)	0.010 (4)
C2A	0.021 (4)	0.032 (5)	0.020 (4)	0.006 (4)	0.002 (3)	0.009 (4)
C2B	0.022 (5)	0.033 (5)	0.027 (5)	0.008 (4)	0.000 (4)	0.007 (4)
C2C	0.015 (4)	0.028 (5)	0.030 (5)	0.001 (3)	0.003 (3)	0.015 (4)
C3A	0.044 (6)	0.036 (5)	0.028 (5)	0.012 (5)	0.000 (4)	0.014 (4)
C3B	0.022 (5)	0.032 (5)	0.035 (6)	−0.003 (4)	0.001 (4)	0.007 (4)
C3C	0.021 (5)	0.047 (6)	0.031 (5)	0.013 (4)	0.010 (4)	0.020 (5)
C4A	0.050 (6)	0.047 (6)	0.030 (5)	0.018 (5)	0.021 (5)	0.023 (5)
C4B	0.038 (6)	0.034 (5)	0.030 (5)	0.003 (4)	0.006 (4)	0.007 (4)
C4C	0.025 (5)	0.023 (5)	0.042 (6)	−0.004 (4)	0.001 (4)	0.022 (4)
C5A	0.031 (5)	0.050 (6)	0.046 (7)	0.014 (5)	0.020 (5)	0.027 (5)
C5B	0.033 (5)	0.034 (5)	0.036 (6)	0.013 (4)	0.010 (4)	0.004 (4)
C5C	0.041 (6)	0.020 (4)	0.041 (6)	0.008 (4)	0.005 (5)	0.014 (4)
C6A	0.015 (4)	0.045 (6)	0.042 (6)	0.007 (4)	0.007 (4)	0.018 (5)
C6B	0.027 (5)	0.031 (5)	0.026 (5)	0.012 (4)	0.009 (4)	0.008 (4)
C6C	0.029 (5)	0.019 (4)	0.031 (5)	0.002 (4)	0.010 (4)	0.011 (4)
C11	0.028 (5)	0.069 (8)	0.023 (5)	0.014 (5)	0.007 (4)	0.014 (5)
C11A	0.016 (4)	0.024 (4)	0.016 (4)	0.001 (3)	0.005 (3)	0.009 (3)
C11B	0.010 (4)	0.021 (4)	0.027 (5)	0.007 (3)	0.004 (3)	0.012 (3)
C11C	0.008 (4)	0.029 (5)	0.028 (5)	0.006 (3)	−0.002 (3)	0.016 (4)
C12	0.021 (5)	0.048 (6)	0.034 (6)	0.002 (4)	0.006 (4)	0.014 (5)

### (II) Poly[hexakis(µ2-4-chloro-3-nitrobenzoato-κ2O:O')bis(dimethyl sulfoxide-κO)dierbium(III)] Geometric parameters (Å, º)

**Table:**

Er1—O11	2.306 (7)	C1B—C6B	1.419 (14)
Er1—O11C	2.312 (8)	C1B—C11B	1.496 (13)
Er1—O12A	2.317 (7)	C1C—C2C	1.398 (13)
Er1—O12B	2.239 (6)	C1C—C6C	1.387 (14)
Er1—O12C^i^	2.287 (6)	C1C—C11C	1.507 (14)
Er1—O11A^ii^	2.300 (6)	C2A—C3A	1.386 (14)
Er1—O11B^ii^	2.348 (6)	C2B—C3B	1.390 (15)
Cl4A—C4A	1.729 (13)	C2C—C3C	1.354 (16)
Cl4B—C4B	1.714 (11)	C3A—C4A	1.361 (17)
Cl4C—C4C	1.730 (11)	C3B—C4B	1.383 (15)
S1—O11	1.514 (8)	C3C—C4C	1.391 (15)
S1—C11	1.785 (10)	C4A—C5A	1.396 (16)
S1—C12	1.772 (11)	C4B—C5B	1.391 (15)
O11A—C11A	1.274 (11)	C4C—C5C	1.367 (15)
O11B—C11B	1.255 (10)	C5A—C6A	1.368 (16)
O11C—C11C	1.255 (11)	C5B—C6B	1.394 (15)
O12A—C11A	1.250 (10)	C5C—C6C	1.391 (15)
O12B—C11B	1.249 (10)	C2A—H2A	0.9500
O12C—C11C	1.271 (12)	C2B—H2B	0.9500
O31A—N3A	1.206 (17)	C2C—H2C	0.9500
O31B—N3B	1.151 (16)	C5A—H5A	0.9500
O31C—N3C	1.191 (16)	C5B—H5B	0.9500
O32A—N3A	1.229 (14)	C5C—H5C	0.9500
O32B—N3B	1.13 (2)	C6A—H6A	0.9500
O32C—N3C	1.188 (15)	C6B—H6B	0.9500
N3A—C3A	1.480 (16)	C6C—H6C	0.9500
N3B—C3B	1.474 (14)	C11—H111	0.9800
N3C—C3C	1.481 (14)	C11—H112	0.9800
C1A—C2A	1.380 (13)	C11—H113	0.9800
C1A—C6A	1.386 (14)	C12—H121	0.9800
C1A—C11A	1.524 (13)	C12—H122	0.9800
C1B—C2B	1.369 (13)	C12—H123	0.9800
			
O11—Er1—O11C	72.5 (3)	N3C—C3C—C2C	117.7 (9)
O11—Er1—O12A	74.7 (2)	N3C—C3C—C4C	120.7 (10)
O11—Er1—O12B	80.6 (2)	C2C—C3C—C4C	121.5 (9)
O11—Er1—O12C^i^	77.0 (3)	Cl4A—C4A—C3A	124.2 (9)
O11—Er1—O11A^ii^	140.9 (3)	Cl4A—C4A—C5A	117.3 (9)
O11—Er1—O11B^ii^	143.3 (2)	C3A—C4A—C5A	118.5 (11)
O11C—Er1—O12A	145.4 (2)	Cl4B—C4B—C3B	124.4 (8)
O11C—Er1—O12B	84.1 (2)	Cl4B—C4B—C5B	116.3 (8)
O11C—Er1—O12C^i^	94.7 (2)	C3B—C4B—C5B	119.3 (10)
O11A^ii^—Er1—O11C	73.9 (2)	Cl4C—C4C—C3C	121.1 (8)
O11B^ii^—Er1—O11C	130.3 (2)	Cl4C—C4C—C5C	119.7 (9)
O12A—Er1—O12B	80.0 (2)	C3C—C4C—C5C	119.2 (10)
O12A—Er1—O12C^i^	88.4 (2)	C4A—C5A—C6A	121.0 (10)
O11A^ii^—Er1—O12A	130.5 (2)	C4B—C5B—C6B	120.5 (10)
O11B^ii^—Er1—O12A	83.5 (2)	C4C—C5C—C6C	120.1 (10)
O12B—Er1—O12C^i^	156.8 (2)	C1A—C6A—C5A	119.9 (9)
O11A^ii^—Er1—O12B	76.7 (2)	C1B—C6B—C5B	119.3 (9)
O11B^ii^—Er1—O12B	124.6 (2)	C1C—C6C—C5C	120.3 (9)
O11A^ii^—Er1—O12C^i^	125.3 (2)	O11A—C11A—O12A	127.7 (8)
O11B^ii^—Er1—O12C^i^	73.2 (2)	O11A—C11A—C1A	116.0 (7)
O11A^ii^—Er1—O11B^ii^	75.2 (2)	O12A—C11A—C1A	116.3 (8)
O11—S1—C11	103.9 (5)	O11B—C11B—O12B	121.6 (8)
O11—S1—C12	106.0 (5)	O11B—C11B—C1B	119.8 (8)
C11—S1—C12	99.3 (5)	O12B—C11B—C1B	118.6 (8)
Er1—O11—S1	133.1 (4)	O11C—C11C—O12C	123.6 (9)
Er1^ii^—O11A—C11A	140.3 (6)	O11C—C11C—C1C	118.1 (8)
Er1^ii^—O11B—C11B	110.9 (5)	O12C—C11C—C1C	118.3 (8)
Er1—O11C—C11C	113.9 (6)	C1A—C2A—H2A	120.00
Er1—O12A—C11A	132.8 (6)	C3A—C2A—H2A	120.00
Er1—O12B—C11B	172.3 (6)	C1B—C2B—H2B	120.00
Er1^i^—O12C—C11C	128.2 (6)	C3B—C2B—H2B	120.00
O31A—N3A—O32A	124.3 (12)	C1C—C2C—H2C	120.00
O31A—N3A—C3A	118.5 (10)	C3C—C2C—H2C	120.00
O32A—N3A—C3A	117.1 (12)	C4A—C5A—H5A	119.00
O31B—N3B—O32B	118.3 (13)	C6A—C5A—H5A	120.00
O31B—N3B—C3B	120.3 (12)	C4B—C5B—H5B	120.00
O32B—N3B—C3B	121.2 (11)	C6B—C5B—H5B	120.00
O31C—N3C—O32C	124.1 (12)	C4C—C5C—H5C	120.00
O31C—N3C—C3C	116.7 (11)	C6C—C5C—H5C	120.00
O32C—N3C—C3C	119.2 (10)	C1A—C6A—H6A	120.00
C2A—C1A—C6A	119.6 (9)	C5A—C6A—H6A	120.00
C2A—C1A—C11A	120.3 (8)	C1B—C6B—H6B	120.00
C6A—C1A—C11A	120.1 (8)	C5B—C6B—H6B	120.00
C2B—C1B—C6B	119.5 (9)	C1C—C6C—H6C	120.00
C2B—C1B—C11B	121.6 (8)	C5C—C6C—H6C	120.00
C6B—C1B—C11B	118.9 (8)	S1—C11—H111	109.00
C2C—C1C—C6C	119.0 (9)	S1—C11—H112	109.00
C2C—C1C—C11C	120.7 (8)	S1—C11—H113	109.00
C6C—C1C—C11C	120.3 (8)	H111—C11—H112	109.00
C1A—C2A—C3A	119.6 (9)	H111—C11—H113	110.00
C1B—C2B—C3B	120.6 (9)	H112—C11—H113	110.00
C1C—C2C—C3C	119.8 (9)	S1—C12—H121	109.00
N3A—C3A—C2A	115.0 (10)	S1—C12—H122	109.00
N3A—C3A—C4A	123.6 (10)	S1—C12—H123	109.00
C2A—C3A—C4A	121.4 (10)	H121—C12—H122	110.00
N3B—C3B—C2B	116.5 (9)	H121—C12—H123	109.00
N3B—C3B—C4B	122.7 (10)	H122—C12—H123	109.00
C2B—C3B—C4B	120.7 (9)		
			
O11C—Er1—O11—S1	123.8 (6)	O31C—N3C—C3C—C2C	−58.0 (16)
O12A—Er1—O11—S1	−67.4 (6)	C6A—C1A—C11A—O11A	−20.4 (13)
O12B—Er1—O11—S1	−149.5 (6)	C2A—C1A—C11A—O11A	158.7 (9)
O12C^i^—Er1—O11—S1	24.6 (5)	C2A—C1A—C11A—O12A	−20.0 (13)
O11A^ii^—Er1—O11—S1	155.6 (4)	C2A—C1A—C6A—C5A	−0.8 (16)
O11B^ii^—Er1—O11—S1	−11.7 (8)	C11A—C1A—C6A—C5A	178.3 (10)
O11—Er1—O11C—C11C	−136.0 (7)	C11A—C1A—C2A—C3A	−175.0 (9)
O12A—Er1—O11C—C11C	−155.2 (6)	C6A—C1A—C2A—C3A	4.1 (15)
O12B—Er1—O11C—C11C	142.1 (6)	C6A—C1A—C11A—O12A	160.9 (9)
O12C^i^—Er1—O11C—C11C	−61.2 (6)	C2B—C1B—C11B—O12B	−4.0 (14)
O11A^ii^—Er1—O11C—C11C	64.2 (6)	C2B—C1B—C11B—O11B	177.2 (9)
O11B^ii^—Er1—O11C—C11C	10.6 (7)	C6B—C1B—C11B—O11B	−4.2 (14)
O11—Er1—O12A—C11A	−102.6 (8)	C11B—C1B—C6B—C5B	−177.4 (9)
O11C—Er1—O12A—C11A	−83.6 (8)	C2B—C1B—C6B—C5B	1.2 (15)
O12B—Er1—O12A—C11A	−19.8 (7)	C6B—C1B—C2B—C3B	0.5 (16)
O12C^i^—Er1—O12A—C11A	−179.6 (8)	C11B—C1B—C2B—C3B	179.1 (10)
O11A^ii^—Er1—O12A—C11A	43.0 (8)	C6B—C1B—C11B—O12B	174.7 (9)
O11B^ii^—Er1—O12A—C11A	107.2 (8)	C6C—C1C—C11C—O11C	−18.6 (13)
O11—Er1—O12C^i^—C11C^i^	−162.8 (8)	C2C—C1C—C11C—O11C	160.3 (8)
O11C—Er1—O12C^i^—C11C^i^	126.4 (8)	C2C—C1C—C11C—O12C	−18.5 (12)
O12A—Er1—O12C^i^—C11C^i^	−88.1 (8)	C11C—C1C—C2C—C3C	−179.3 (9)
O12B—Er1—O12C^i^—C11C^i^	−147.7 (7)	C6C—C1C—C11C—O12C	162.7 (9)
O11—Er1—O11A^ii^—C11A^ii^	85.7 (10)	C2C—C1C—C6C—C5C	1.5 (14)
O11C—Er1—O11A^ii^—C11A^ii^	117.2 (10)	C6C—C1C—C2C—C3C	−0.5 (14)
O12A—Er1—O11A^ii^—C11A^ii^	−34.5 (11)	C11C—C1C—C6C—C5C	−179.6 (9)
O12B—Er1—O11A^ii^—C11A^ii^	29.6 (9)	C1A—C2A—C3A—C4A	−5.3 (17)
O11—Er1—O11B^ii^—C11B^ii^	−118.1 (6)	C1A—C2A—C3A—N3A	173.0 (10)
O11C—Er1—O11B^ii^—C11B^ii^	123.2 (6)	C1B—C2B—C3B—C4B	−3.0 (17)
O12A—Er1—O11B^ii^—C11B^ii^	−64.8 (6)	C1B—C2B—C3B—N3B	177.8 (10)
O12B—Er1—O11B^ii^—C11B^ii^	8.3 (7)	C1C—C2C—C3C—N3C	177.0 (9)
C11—S1—O11—Er1	154.8 (6)	C1C—C2C—C3C—C4C	−0.4 (15)
C12—S1—O11—Er1	−101.1 (6)	C2A—C3A—C4A—Cl4A	−174.7 (9)
Er1^ii^—O11A—C11A—O12A	−5.6 (16)	C2A—C3A—C4A—C5A	2.9 (18)
Er1^ii^—O11A—C11A—C1A	175.9 (7)	N3A—C3A—C4A—C5A	−175.2 (11)
Er1^ii^—O11B—C11B—O12B	−0.5 (11)	N3A—C3A—C4A—Cl4A	7.2 (18)
Er1^ii^—O11B—C11B—C1B	178.3 (7)	N3B—C3B—C4B—C5B	−177.1 (11)
Er1—O11C—C11C—O12C	14.1 (11)	C2B—C3B—C4B—Cl4B	−178.6 (9)
Er1—O11C—C11C—C1C	−164.6 (6)	C2B—C3B—C4B—C5B	3.8 (17)
Er1—O12A—C11A—O11A	−27.7 (14)	N3B—C3B—C4B—Cl4B	0.6 (17)
Er1—O12A—C11A—C1A	150.8 (6)	N3C—C3C—C4C—Cl4C	1.6 (14)
Er1^i^—O12C—C11C—O11C	95.6 (9)	N3C—C3C—C4C—C5C	−177.1 (10)
Er1^i^—O12C—C11C—C1C	−85.7 (9)	C2C—C3C—C4C—Cl4C	178.9 (8)
O32A—N3A—C3A—C2A	−150.4 (12)	C2C—C3C—C4C—C5C	0.2 (15)
O31A—N3A—C3A—C4A	−154.6 (14)	Cl4A—C4A—C5A—C6A	178.2 (10)
O32A—N3A—C3A—C4A	27.9 (18)	C3A—C4A—C5A—C6A	0.5 (19)
O31A—N3A—C3A—C2A	27.2 (17)	Cl4B—C4B—C5B—C6B	−179.9 (9)
O32B—N3B—C3B—C4B	−5 (2)	C3B—C4B—C5B—C6B	−2.0 (17)
O31B—N3B—C3B—C2B	−0.8 (19)	Cl4C—C4C—C5C—C6C	−177.9 (8)
O31B—N3B—C3B—C4B	−179.9 (14)	C3C—C4C—C5C—C6C	0.9 (16)
O32B—N3B—C3B—C2B	174.1 (16)	C4A—C5A—C6A—C1A	−1.5 (18)
O32C—N3C—C3C—C4C	−62.2 (16)	C4B—C5B—C6B—C1B	−0.5 (16)
O31C—N3C—C3C—C4C	119.4 (14)	C4C—C5C—C6C—C1C	−1.7 (16)
O32C—N3C—C3C—C2C	120.3 (13)		

Symmetry codes: (i) −*x*, −*y*+1, −*z*+1; (ii) −*x*+1, −*y*+1, −*z*+1.

### (II) Poly[hexakis(µ2-4-chloro-3-nitrobenzoato-κ2O:O')bis(dimethyl sulfoxide-κO)dierbium(III)] Hydrogen-bond geometry (Å, º)

**Table:**

*D*—H···*A*	*D*—H	H···*A*	*D*···*A*	*D*—H···*A*
C2*A*—H2*A*···S1	0.95	2.86	3.743 (10)	155
C2*B*—H2*B*···O11	0.95	2.56	3.298 (13)	135
C11—H111···Cl4*A*^iii^	0.98	2.79	3.486 (11)	129
C12—H123···O32*A*^iv^	0.98	2.44	3.376 (15)	158

Symmetry codes: (iii) −*x*+1, −*y*+1, −*z*; (iv) −*x*, −*y*+1, −*z*.
